# Arginine Vasopressin-Independent Mechanism of Impaired Water Excretion in a Patient with Sarcoidosis Complicated by Central Diabetes Insipidus and Glucocorticoid Deficiency

**DOI:** 10.1155/2011/145856

**Published:** 2011-07-31

**Authors:** Katsunobu Yoshioka, Nagaaki Tanaka, Keiko Yamagami, Takeshi Inoue, Masayuki Hosoi

**Affiliations:** ^1^Department of Internal Medicine, Osaka City Sumiyoshi Hospital, 1-2-16, Higashikagaya, Suminoeku, Osaka City, Osaka 559-0012, Japan; ^2^Department of Internal Medicine, Osaka City Juso Hospital, Osaka 532-0034, Japan; ^3^Department of Endocrinology and Metabolism, Osaka City General Hospital, Osaka 534-0021, Japan; ^4^Department of Pathology, Osaka City General Hospital, Osaka 534-0021, Japan

## Abstract

A 28-year-old man was admitted to our hospital because of reduced livido and increased fatigability. Four months before admission, he noticed polyuria, which was gradually relieved by admission. Magnetic resonance imaging revealed enhancing lesion centrally in the pituitary stalk. Biopsy from the skin revealed noncaseating granuloma composed of epithelioid cells, and a diagnosis of sarcoidosis was made. Although plasma arginine vasopressin (AVP) was undetectable after administration of hypertonic saline, urinary output was within normal range (1.5 to 2.2 L/day). The urine osmolality became above plasma levels during the hypertonic saline test. Hormonal provocative tests revealed partial glucocorticoid deficiency. Soon after the glucocorticoid therapy was begun, moderate polyuria (from 3.5–4.0 liters daily) occurred. At this time, plasma AVP was undetectable, and urine osmolality was consistently below plasma levels during the hypertonic saline test. In conclusion, we showed in human study that masked diabetes insipidus could be mediated by AVP-independent mechanisms.

## 1. Introduction

It is well recognized that polyuria is relieved in patients with central diabetes insipidus (CDI), when they also experience glucocorticoid (GC) deficiency; this phenomenon is known as masked diabetes insipidus (DI). However, the mechanisms mediating masked DI are not clear. GC deficiency is known to increase levels of arginine vasopressin (AVP) [1] and exaggerate the action of AVP [2], suggesting that masked DI is mediated mainly by AVP-dependent mechanisms. On the other hand, in animal models of DI (Brattleboro rats; hereditary hypothalamic AVP-deficient rats), GC deficiency caused impaired water excretion [3], suggesting that AVP-independent mechanisms are also involved. To elucidate the role of AVP in masked DI, it is essential to evaluate plasma AVP levels using a hypertonic saline infusion test before and after GC replacement. However, such cases are seldom reported [4], most likely because clinicians do not suspect DI during nonpolyuric periods. Thus, the role of AVP in masked DI is still controversial.

Here, we report a patient with sarcoidosis complicated by CDI and GC deficiency in whom a hypertonic saline infusion test was done before and after GC replacement. Results showed that masked DI could be mediated solely by AVP-independent mechanisms.

## 2. Case Report

A 28-year-old man was admitted to our hospital because of reduced livido and increased fatigability. He had been in good health until 1 year earlier, when he noticed reduced livido. Four months before admission, he noticed frequent headaches, thirst, polydipsia, and polyuria. Because he was voiding urine every hour, he visited his family doctor. Magnetic resonance imaging (MRI) revealed a suprasellar mass, and he was thus referred to our hospital for further evaluation and treatment. Although polydipsia and polyuria had been gradually relieved by the time of admission, he became aware of increased fatigability. 

**Figure 1:**
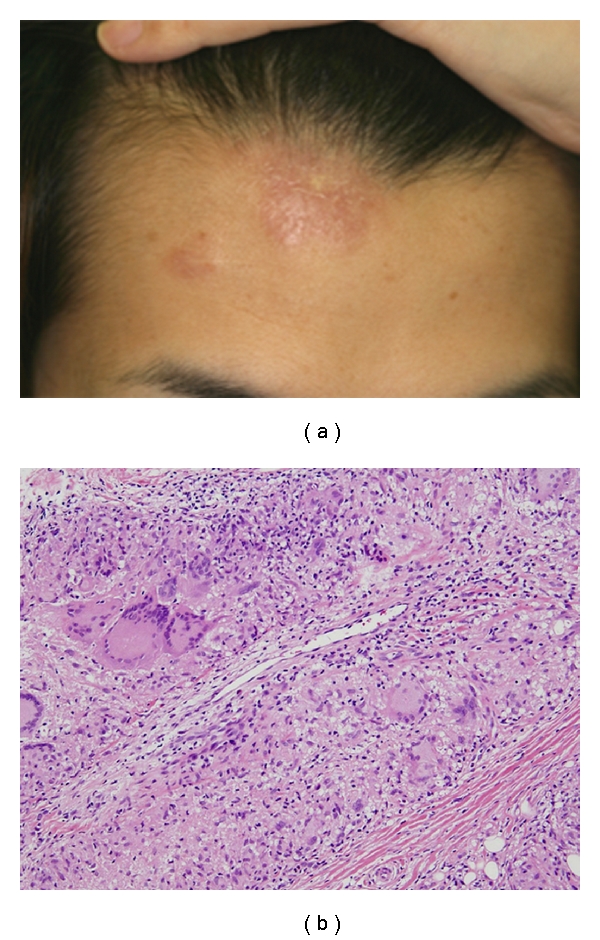
Photograph of the skin of the forehead (a) and pathological finding of the skin biopsy (b). Noncaseating granuloma composed of epithelioid cells and multinucleated giant cells were present.

At the time of admission, he was 176 cm tall and weighed 72 kg. His temperature was 37.6°C, pulse rate was 60/min, and blood pressure was 110/60 mmHg. No goiter or enlargement of lymph nodes was noted. Axillar and pubic hair were lost and there was palpable erythematous rush on his forehead (Figure 1(a)). The remainder of the physical examination was unremarkable.

Laboratory results revealed the following results: blood urea nitrogen 9.6 mg/dL, creatinine 0.79 mg/dL, serum sodium 141 mEq/l, potassium 4.0 mEq/l, chloride 100 mEq/l, calcium 9.4 mg/dL, phosphate 3.7 mg/dL, fasting plasma glucose 80 mg/dL, and C-reactive protein 0.07 mg/dL. Endocrinologic data revealed a mild decrease in free T4 level (0.6 ng/dL) with normal thyroid-stimulating hormone (TSH) level (0.9106 *μ*U/mL) and decreased cortisol (4.7 *μ*g/mL), adrenocorticotropic hormone (ACTH; 6 pg/mL), luteinizing hormone (LH; 0.2 mIU/mL), follicle stimulating hormone (FSH; 1.3 mIU/mL), and testosterone (0.05 ng/mL) levels. Urinary cortisol was undetectable. Plasma osmolality was 288 mOsm/kgH_2_O, with a urinary osmolality of 239 mOsm/kgH_2_O, and arginine vasopressin (AVP) was undetectable (<0.15 pg/mL). Urinary output was from 1.5 to 2.2 L/day.

**Figure 2:**
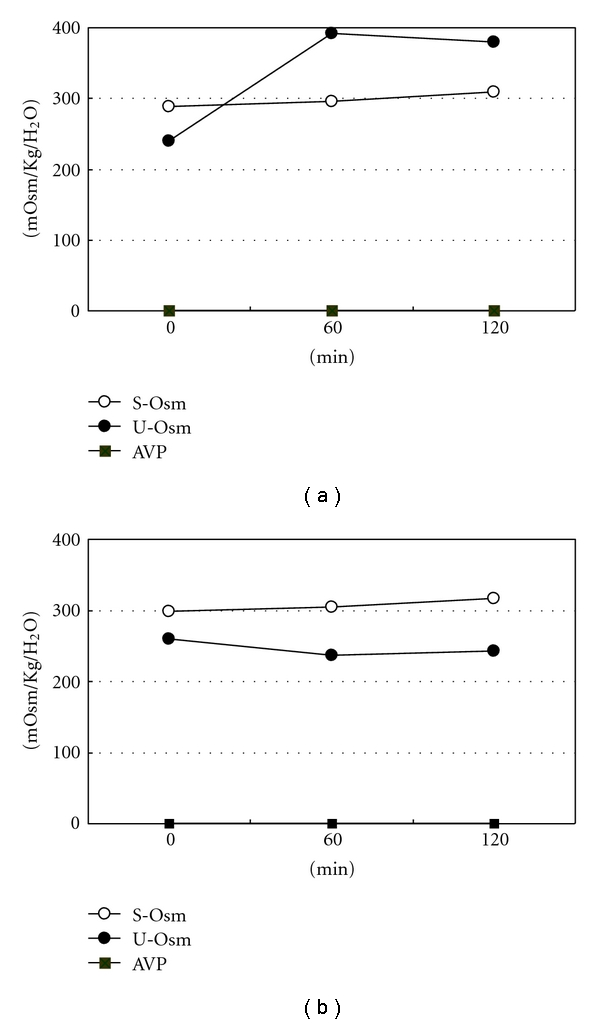
Hypertonic saline test before (a) and after (b) glucocorticoid therapy. Before therapy, although plasma AVP was undetectable, urine became concentrated. After therapy, the hypertonic saline test failed to concentrate the urine and AVP was undetectable.

**Figure 3:**
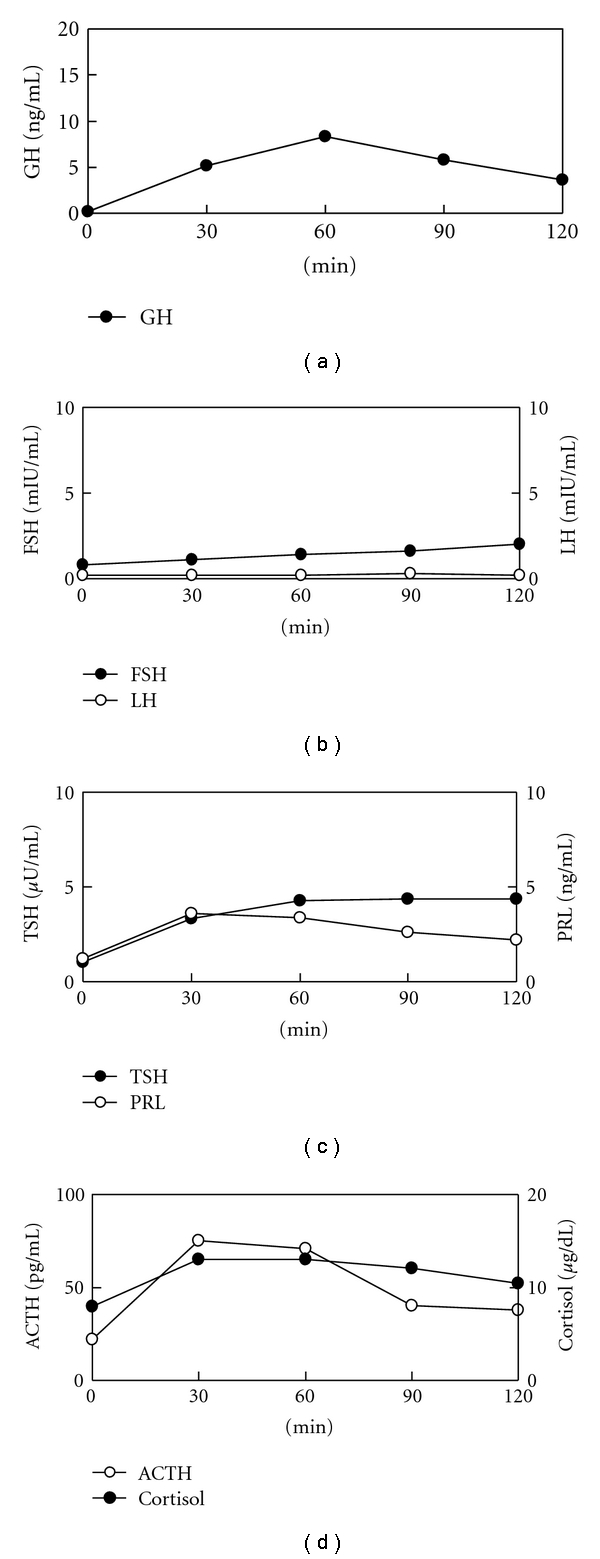
Hormonal provocative test results.

His clinical course and laboratory findings led us to suspect CDI with anterior pituitary dysfunction. We thus performed hormonal provocative tests. The patient was given an infusion of 5% saline at a rate of 0.05 mL/kg for 120 min. Although plasma AVP was undetectable before and after administration of hypertonic saline, urine osmolality (391 mOsm/kgH_2_O) increased above the plasma levels (296 mOsm/kgH_2_O) (Figure 2(a)). Hormonal provocative tests revealed that growth hormone (GH) response to GH-releasing hormone was normal, TSH response to TSH-releasing hormone was low and blunted, and FSH and LH response to LH-releasing hormone were low and blunted (Figure 3). Although ACTH response to corticotrophin-releasing hormone (CRH) was normal, cortisol response to CRH was low (maximum 13.0 *μ*g/mL). Cortisol response to rapid ACTH stimulation test was low (maximum 15.9 *μ*g/mL). Results of these hormonal tests were compatible with the diagnosis of CDI with partial GC deficiency. 

**Figure 4:**
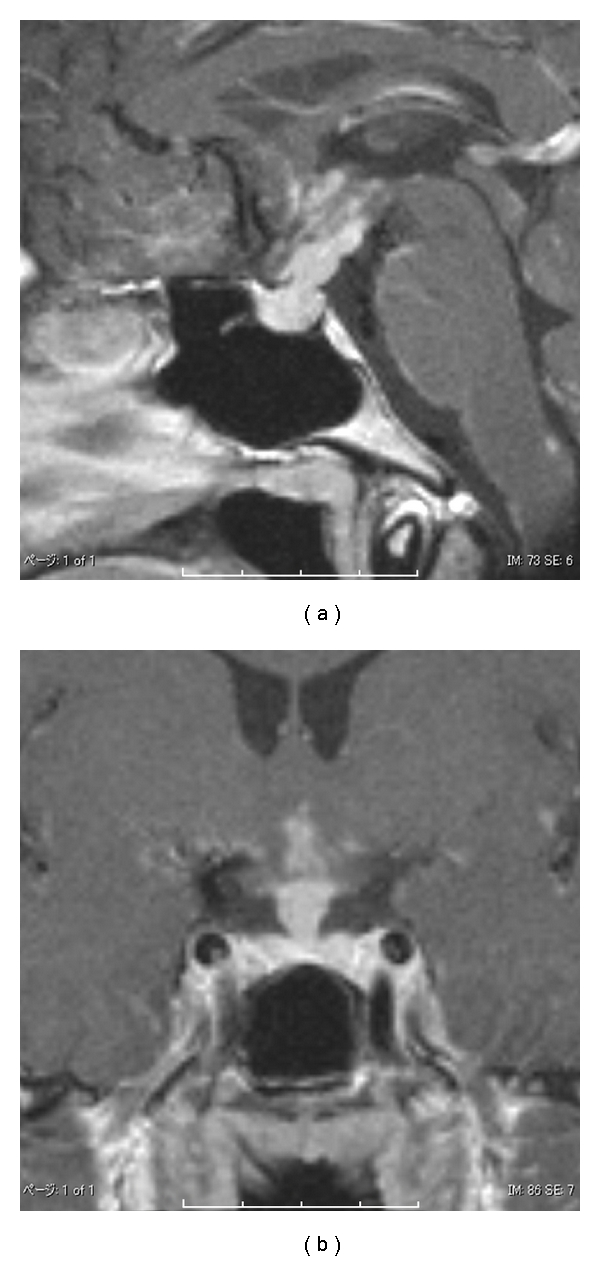
T1-weighted MRI of the sella turnica after administration of gadolinium revealed enhancing lesion centrally in the pituitary stalk, which spread continuously to the bottom of the third ventricle, and high intensity of posterior lobe was lost.

MRI revealed enhancing lesion centrally in the pituitary stalk, which spread continuously to the bottom of the third ventricle, and the high intensity of the posterior lobe was lost, compatible with the diagnosis of CDI (Figure 4). Considering the patient's age, we first suspected of neurohypophysial germinoma. However, this was unlikely because cerebrospinal fluid was negative for malignant cells, and *β*-human chorionic gonadotropin (HCG) levels were normal. Next, we suspected sarcoidosis because chest X-ray revealed hilar enlargement. A biopsy from the skin of the forehead was performed, which revealed noncaseating granuloma composed of epithelioid cells (Figure 1(b)). Multinucleated giant cells were also present. Thus, the diagnosis of sarcoidosis was made. 

**Figure 5:**
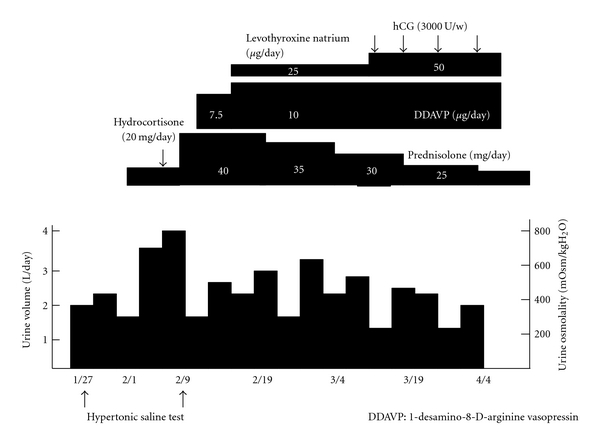
Clinical course.

While waiting for histologic confirmation of biopsy results, hydrocortisone replacement was begun with a dose of 20 mg daily. Soon after the replacement therapy was begun, moderate polyuria (3.5–4.0 liters daily) occurred (Figure 2(b)). After the diagnosis of sarcoidosis was confirmed, he was treated with 40 mg of prednisolone and his general condition improved. The hypertonic saline test was repeated while he was taking 40 mg of prednisolone. Plasma AVP was undetectable during the hypertonic saline test and urine osmolality was consistently below plasma levels, which was compatible with a diagnosis of CDI (Figure 5). He was treated with 1-desamino-8-D-arginine vasopressin (dDAVP).

MRI following prednisolone therapy revealed a reduction in the size of the affected lesion. However, his pituitary function remained unchanged, and he is currently being treated with prednisolone, levothyroxine natrium, HCG, and dDAVP.

## 3. Discussion

It is difficult to suspect the existence of “masked DI” before starting GC replacement therapy because patients do not complain of characteristic symptoms of DI such as polyuria and polydipsia. The present patient's symptoms of polyuria and polydipsia gradually decreased while fatigability gradually increased, which prompted us to suspect the likelihood of masked DI. We thus performed the hypertonic saline test even though he did not complain of overt polyuria on admission. Although plasma AVP was undetectable before and after administration of hypertonic saline, urine osmolality increased above plasma levels. Radioimmunoassay for AVP is sensitive enough to detect a small quantity of circulating AVP, suggesting that kidneys can concentrate the urine even if circulating AVP is completely deficient. After starting GC replacement, plasma AVP was undetectable before and after administration of hypertonic saline. However, urine osmolality was consistently below plasma levels. These observations suggest that GC deficiency can cause impaired water excretion, which is not mediated by AVP-dependent mechanisms. 

Although AVP-deficient rat models clearly show that GC deficiency can cause impaired water excretion independent of AVP, the underlying mechanisms are still unclear. In one study, adrenalectomized Brattleboro rats experienced a rise in water permeability of the distal tubules, suggesting that one of the mechanisms might be direct action of GC on renal tubules [5]. On the other hand, GC dilates both afferent and efferent resistances and results in an increase in glomerular filtration rate (GFR), by which GC increases the rate of water flow into the nephron [6]. Thus, GC-induced increase in water excretion may be secondary to an elevation in GFR. GC has been reported to directly stimulate the production of atrial natriuretic polypeptide (ANP) [7] and potentiate the action of ANP [8], resulting in increase in GFR. Furthermore, GC deficiency was associated with a significantly lower cardiac index [9]. Therefore, GC-induced increase in water excretion may be secondary to changes in systemic and renal hemodynamics as well as direct actions on the tubules. Unfortunately, these hemodynamic studies were not done in the present case, although systemic blood pressure and serum creatinine levels were unchanged. Thus, the AVP-independent mechanisms by which GC deficiency caused impaired water excretion in the present case are unknown. 

After starting GC therapy, the diagnosis of CDI became certain based on the results of the hypertonic saline infusion test. However, the degree of polyuria (3.5–4 L/d) was moderate, and maximum urine osmolality was not extremely low (259 mOsm/kgH_2_O) despite undetectable AVP. However, we performed the hypertonic saline test soon after starting GC therapy and it is possible that it takes more time until GC completely restores the diluting ability of urine. Not only GC deficiency but also mineralcorticoid (MC) deficiency is involved in the impaired water excretion in adrenalectomized Brattleboro rats. Thus, MC deficiency could be one reason that the degree of polyuria was moderate even after the GC replacement. We did not check the aldosterone level in this study because secretion of aldosterone is usually preserved in patients with central adrenal deficiency.

Sarcoidosis is a systemic disorder of unknown cause that is characterized pathologically by noncaseating granuloma. Although involvement of sarcoidosis in the central nervous system is rare (approximately 5%), the hypothalamus is one of the most affected organs in neurosarcoidosis. Thus, various degrees of secondary hypopituitarism can occur. Among them, CDI is the most reported endocrine abnormality, occurring in 25% of patients with neurosarcoidosis [10]. Although any degree of anterior pituitary insufficiency can also occur, gonadotropin deficiency is most common. In the present case, endocrinologic manifestations (hypogonadism and DI) were the first clinical symptoms of sarcoidosis. Five similar cases have been reported, in which CDI and hypogonadism were the first clinical presentations of neurosarcoidosis [11]. Thus, endocrinologic manifestations could be the first clinical presentations of neurosarcoidosis. It has been reported that 97% of patients with neurosarcoidosis have systemic involvement of sarcoidosis even if they are asymptomatic [12]. Furthermore, considering that other hypothalamic lesions causing CDI such as Wegener's granulomatosis [13] and IgG4-related sclerosing disease [14] usually have extra-hypothalamic lesions, clinicians should search for systemic involvement to establish the diagnosis if patients are otherwise asymptomatic. In the present case, careful observation of the skin prompted us to obtain a biopsy specimen, which led to proper diagnosis.

In summary, we report on a patient with sarcoidosis complicated by CDI and GC deficiency, whose urine became concentrated after the administration of hypertonic saline although AVP was undetectable. However, following GC therapy, hypertonic saline failed to concentrate the urine. In conclusion, we showed in human study that masked diabetes insipidus could be mediated solely by AVP-independent mechanisms.
